# A Novel Nomogram Based on Imaging Biomarkers of Shear Wave Elastography, Angio Planewave Ultrasensitive Imaging, and Conventional Ultrasound for Preoperative Prediction of Malignancy in Patients with Breast Lesions

**DOI:** 10.3390/diagnostics13030540

**Published:** 2023-02-02

**Authors:** Guoqiang Guo, Jiaping Feng, Chunchun Jin, Xuehao Gong, Yihao Chen, Sihan Chen, Zhanghong Wei, Huahua Xiong, Jianghao Lu

**Affiliations:** 1 Department of Ultrasound, Shenzhen Second People’s Hospital, The First Affiliated Hospital of Shenzhen University, Sungang West Road 3002, Futian District, Shenzhen 518025, China; 2 Graduate School, Guangzhou Medical University, Guangzhou 510180, China; 3 Department of Ultrasound, Heping People’s Hospital, Dongshan Road 10, Yangming Town, Heping County, Heyuan 517299, China

**Keywords:** breast neoplasms, shear wave elastography, diagnosis, nomogram, ultrasonography

## Abstract

Several studies have demonstrated the difficulties in distinguishing malignant lesions of the breast from benign lesions owing to overlapping morphological features on ultrasound. Consequently, we aimed to develop a nomogram based on shear wave elastography (SWE), Angio Planewave Ultrasensitive imaging (Angio PLUS (AP)), and conventional ultrasound imaging biomarkers to predict malignancy in patients with breast lesions. This prospective study included 117 female patients with suspicious lesions of the breast. Features of lesions were extracted from SWE, AP, and conventional ultrasound images. The least absolute shrinkage and selection operator (Lasso) algorithms were used to select breast cancer-related imaging biomarkers, and a nomogram was developed based on six of the 16 imaging biomarkers. This model exhibited good discrimination (area under the receiver operating characteristic curve (AUC): 0.969; 95% confidence interval (CI): 0.928, 0.989) between malignant and benign breast lesions. Moreover, the nomogram also showed demonstrated good calibration and clinical usefulness. In conclusion, our nomogram can be a potentially useful tool for individually-tailored diagnosis of breast tumors in clinical practice.

## 1. Introduction

Breast cancer is the most common neoplasm and leading cause of cancer-related deaths among the female population worldwide [1]. In addition, early and accurate diagnosis is of great significance in improving the prognosis of breast cancer patients [2]. Conventional ultrasound (US) is an important screening modality for early detection of breast lesions owing to its cost-effectiveness and the advantage of being radiation-free [3]. However, several studies have demonstrated the difficulties in distinguishing malignant lesions of the breast from benign lesions with overlapping morphological features on ultrasound [4,5,6]. In the era of precision medicine, multimodal ultrasound diagnosis of breast tumors has become the general trend. Moreover, shear wave elastography (SWE) and Angio Planewave ultrasensitive imaging (Angio PLUS (AP)) are new ultrasound techniques that have recently been used in the diagnosis of breast tumors [7,8,9]. 

SWE is an imaging technique that excites the lesional tissue by pushing ultrasound radiation force to generate shear waves. The ultrasound imaging system then captures these waves and encodes them to a color map overlying the grey-scale B-mode image [10]. The properties of malignant lesions tend to be heterogeneous and stiffer than benign ones on the color map. Another advantage of SWE is that it can evaluate the stiffness of lesions quantitatively by measuring elasticity parameters in region of interest (ROI), such as Elasticity maximum (E-max), Elasticity mean (E-mean), Elasticity minimum (E-min), and Elasticity standard deviation (E-sd), etc. [11]. Previous studies have demonstrated that regardless of whether the stiffness of lesions is assessed qualitatively or quantitatively, SWE can be used as a supplementary examination method to improve the diagnostic performance of a breast tumor [12,13,14].

Color Doppler Flowing Imaging (CDFI) and power Doppler Imaging (PDI) are widely used to display the number and characteristics of blood vessels within breast lesions. However, several studies have shown that it may be difficult for CDFI and PDI to differentiate the microvasculature within breast lesions [15,16]. AP is a novel doppler technique that can obtain improved microvascular blood flow signals compared to CDFI, by transmitting plane waves rather than a focused ultrasound pulse. Furthermore, it can significantly enhance the spatial resolution of the microvasculature through the three-dimensional wall filter system [17]. 

Stiffness and vascularity are two important and complementary features of breast lesions. Therefore, we hypothesized that additional tools used in adjunct with conventional ultrasound—i.e., SWE and AP—might help improve the diagnostic accuracy for the differentiation of benign and malignant breast tumors. In addition, the nomograms provide clinicians with a quantitative way to determine the probability of disease based on individual characteristics. However, to the best of our knowledge, there is no previous model for differentiating between malignant and benign breast tumors by combining conventional ultrasound, SWE, and AP. Therefore, the aim of our study is to develop a nomogram based on SWE, AP, and conventional ultrasound imaging biomarkers to predict malignancy in patients with breast lesions, which would provide a visible way to quickly obtain a patient’s individual chances of having breast cancer.

## 2. Materials and Methods

### 2.1. Patient Selection

**Figure 1:**
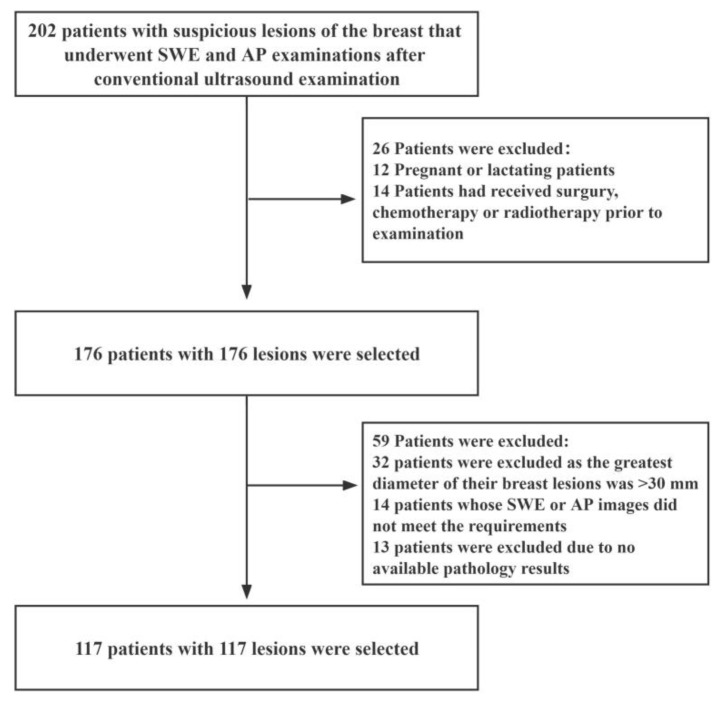
Flow diagram for the patient selection process. AP: Angio Planewave Ultrasensitive Imaging; SWE: Shear Wave Elastography.

This prospective study was approved by the Ethics Committee of The First Affiliated Hospital of Shenzhen University (20220901004). We recruited female patients with suspicious breast lesions between December 2021 and August 2022. The inclusion criteria were as follows: (i) female patients aged 18 years or older; (ii) patients with solid masses in the breast that had undergone complete US, SWE, and AP examinations; (iii) patients scheduled for surgery or ultrasound-guided needle biopsy, and (iv) those with multiple suspicious masses of the breast (in such an event, the most suspicious lesion was selected for further analysis). The exclusion criteria were as follows: (i) pregnant or lactating patients; (ii) patients that had undergone breast surgery, chemotherapy or radiotherapy before the examination; (iii) patients whose radiographic imaging quality did not meet the requirements, and (iv) patients with breast lesions with a maximum diameter greater than 30 mm (as this maximum diameter of breast lesions exceeds the maximum diameter of the sampling frame of SWE). A schematic diagram of the inclusion and exclusion criteria is shown in Figure 1.

### 2.2. Ultrasound Image Acquisition

Conventional ultrasound, AP, and SWE examinations were conducted with a Supersonic Aixplorer system (SuperSonic Imagine, Aix-en-Provence, France) using a 15–4 probe. All examinations were performed by a single sonographer with 6 years of experience in breast imaging. 

Initially, patients were positioned in a supine position with their ipsilateral arm raised, and conventional ultrasound including grey-scale ultrasound and CDFI was performed. Then, when a target lesion was detected, the features of the lesion were clearly depicted according to the fifth edition of Breast Imaging Reporting and Data System (BI-RADS). Regarding AP examination, we applied the abundant gel and minimal pressure with the transducer to avoid obliterating the microvasculature within the lesions. More importantly, we obtained static images and video clips from the layer with the most abundant blood vessels and no noise. In addition, the following were the details of the applied settings: color velocity scale, 2 cm/s; wall filter, medium; frame rate, medium; dynamic range, medium, and turn on flash suppression mode. 

SWE examination was carried out at the largest diameter of the target mass after the US and AP examinations. A large amount of gel and minimal precompression were applied to reduce artifactual stiffness. First, patients were asked to hold their breath for a few seconds to obtain a stable color map. The stiffness range of the color map was 0–180 kPa, which was indicated by a gradient of blue to red colors. Next, we traced the target lesion to measure its stiffness parameters, such as E-max, E-mean, E-min, and E-sd. Lastly, we measured the stiffness ratio (E-ratio) of the lesion compared to the surrounding adipose tissue by placing two ROIs, each of 2 mm diameter, at the same depth as that of the lesion. In addition, all measurements were repeated at least three times, and details about individual parameter definitions are presented in Appendix A.

### 2.3. Ultrasound Image Evaluation 

Two experienced sonographers independently analyzed DICOM-formatted images exported from the Supersonic Explorer system. Additionally, two experienced sonographers were blinded to the pathological examination results. 

**Figure 2:**
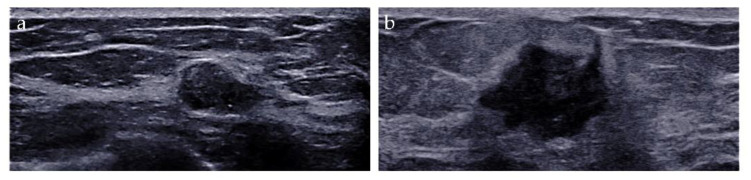
Conventional ultrasound evaluation of breast lesions. (**a**) An irregular-shaped, circumscribed, hypoechoic mass with depth-width ratio < 1, with microcalcifications, absent halo, and no posterior acoustic features. US-guided core needle biopsy revealed this lesion to be an adeno-myoepithelioma. (**b**) An irregular-shaped, non-circumscribed, hypoechoic mass with depth-width ratio < 1, with no microcalcifications, presence of halo, and posterior acoustic enhancement features. Surgical pathology revealed this lesion to be an invasive ductal carcinoma.

The imaging biomarkers of conventional ultrasound were defined as follows: (i) tumor size, maximum; (ii) shape, regular or irregular; (iii) margin, circumscribed or not circumscribed; (iv) depth-width ratio (DWR) > 0.7, present or absent [18]; (v) echo pattern, hypoechoic, isoechoic, hyperechoic, or heterogeneous; (vi) microcalcifications, breast microcalcifications which are tiny calcium deposits that can be seen on ultrasound imaging and range in size from 0.1 to 1.0 mm [19], present or absent; (vii) halo, present or absent, and (viii) posterior features, no posterior acoustic features, enhancement, shadowing, or combined pattern (Figure 2).

**Figure 3:**
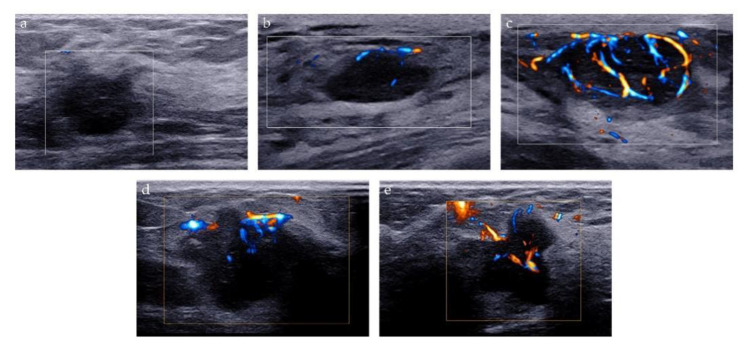
Microvascular distribution pattern of AP. (**a**) Non-vascular pattern; (**b**) Linear or curvilinear pattern; (**c**) Tree-like pattern; (**d**) Root hair-like pattern; (**e**) Crab claw-like pattern.

Two methods were used to analyze the microvascularity of breast lesions on AP examination: Adler classification and microvascular distribution pattern (MVDP). Adler classification is a widely used method to evaluate the blood flow abundance of the tumor. The blood flow signal of the lesion was classified as follows: grade 0 refers to no vascular signal; grade 1, the detection of 1 or 2 small blood vessels with a diameter of <1 mm; grade 2, the detection of 3 or 4 small blood vessels, and grade 3, the detection of more than four blood vessels, or the detection of blood vessels intertwined into a network [20]. MVDP is a method to evaluate the microvascular architecture of lesions and it was used to differentiate between benign and malignant breast lesions in previous studies [21,22,23]. Based on the morphological characteristics of microvessels, MVDP can be classified into five patterns: (i) non-vascular pattern, lack of vessel signals; (ii) linear or curvilinear pattern, single or a few dot-like or linear vessel signals seen in target lesion; (iii) tree-like pattern, vessels which branch proportionally within the lesion; (iv) root hair-like pattern, disordered arrangement of blood vessel signals and less than two dilated and distorted blood vessels around, and (v) crab claw-like pattern, characterized by radial vessels, with many branch vessels in the surrounding area of the lesions (Figure 3).

**Figure 4:**
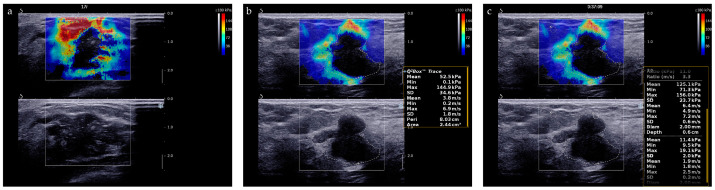
SWE evaluation of breast lesions. (**a**) A breast lesion with “stiff rim” sign on SWE image; (**b**) E-max, E-mean, E-min, and E-sd is automatically calculated by the system after we traced the entire target lesion. (**c**) E-ratio is automatically calculated by the system after two ROIs with a diameter of 2 mm were identified at the same depth as that of the lesion.

The “stiff rim” sign is a color pattern on the SWE image and is of great help for differential diagnosis of breast tumors [24]. The “stiff rim” sign appears locally at the margin of the lesion and forms a closed circle (Figure 4a). As for quantitative evaluation of stiffness of lesions, we recorded the average measurements of E-max, E-mean, E-min, E-sd and E-ratio of breast lesions (Figure 4b,c). Their cut-off values were determined using receiver operating characteristic (ROC) curves analysis.

### 2.4. Histopathological Evaluation

The pathological findings of US-guided core needle biopsy or surgery were interpreted. Accordingly, a pathologist with more than ten years of experience, blinded to the ultrasonographic findings, gave a diagnosis.

### 2.5. Development of the Nomogram

The least absolute shrinkage and selection operator (Lasso) penalty is a regression analysis method that performs both variable selection and regularization [25]. In our study, Lasso penalty was performed to select the most significant features from conventional ultrasound, SWE, and AP imaging biomarkers. Afterward, the nomogram was developed using the independent predictors selected by Lasso to generate a combined imaging biomarker for estimating the probability of breast cancer.

### 2.6. Validation of the Nomogram

Our nomogram was internally validated by using 500 bootstrap samples. Additionally, three main aspects of validation of the nomogram are: discrimination, calibration, and clinical usefulness.

Area under the receiver operator characteristic (ROC) curve (AUC) analysis was conducted to assess the discriminative performance of the nomogram. In addition, the calibration curve was also plotted to evaluate the deviation between the estimated and actual probability [26]. Lastly, decision curve analysis (DCA) was performed to manifest the clinical usefulness of the nomogram by quantifying the net benefit at different probability thresholds [27].

### 2.7. Statistical Analysis

All statistical analyses were conducted using EmpowerStats (R) (X&Y Solutions, Inc., Boston, MA, USA) and R software, version 4.2.1 (http://www.r-project.org accessed on 31 October 2022).

Medians and interquartile ranges described continuous variables that do not conform to the normal distribution, while frequencies and percentages described categorical variables. Next, chi-squared, and Kruskal–Wallis tests were used to assess group differences in categorical and continuous variables, respectively. In addition, Lasso logistic regression was conducted to select the most associated imaging biomarkers of breast cancer; ROC curve analyses were employed to evaluate the diagnostic accuracy of the candidate ultrasound imaging biomarkers. Lastly, the detailed packages of the development and validation of the nomogram in this study are presented in Appendix B.

All statistical tests were two-sided with statistical significance set at a *p* value of <0.05.

## 3. Results

### 3.1. Patient Characteristics

According to pathological diagnosis, 117 patients with 117 lesions were included in the study: 72 patients (61.5%) with benign breast tumors and 45 patients (38.5%) with malignant tumors. The median age of the malignant group was significantly higher than that of the benign group. In addition, the size of lesions in the malignant group was significantly larger than in the benign group (17.5 mm versus 11.4 mm, respectively). 

Conventional ultrasound findings depicting shape, DWR, margin, echo pattern, microcalcification, and posterior features are shown in Table 1.

According to SWE findings, E-max in the malignant group was significantly higher than that in the benign group (139.3 kPa versus 38.2 kPa, respectively). Additionally, the medians of E-mean and E-sd in the malignant group were 40.8 kPa and 27.2 kPa, respectively, which were significantly higher than that in the benign group (18.3 kPa and 6.9 kPa, respectively). However, the E-min showed no significant difference between malignant and benign groups (*p* = 0.944). In the malignant group, there were 34 lesions with “stiff rim” sign (34/45), while in the benign group, there were only 6 lesions (6/72). In terms of AP findings, the blood flow signal of the tumor in the benign group was mainly graded as 0 and 1 (42/72), while the tumor blood flow signal in the malignant group was mainly graded as grade 2 and 3 (39/45). In addition, non-vascular patterns, linear or curvilinear patterns, and tree-like patterns were mainly present in the benign group (61/72). In contrast, root hair-like patterns and crab claw-like patterns were mostly observed in the malignant group (41/45).

Comparisons of multi-imaging biomarkers of ultrasonography between the benign and malignant groups are presented in Table 1.

### 3.2. Pathological Findings

The histopathologic diagnoses of the 117 breast lesions (72 benign and 45 malignant) are shown in Table 2.

### 3.3. Feature Selection and Nomogram Construction

**Figure 5:**
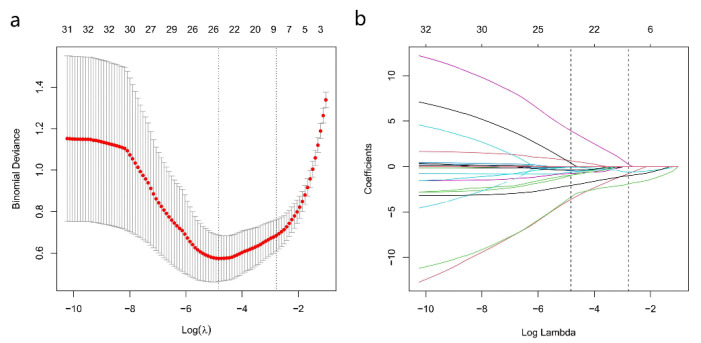
SWE, AP, and Conventional Ultrasound Imaging biomarkers’ selection using the least absolute shrinkage and selection operator (Lasso) algorithm. (**a**) The coefficient convergence graph for the feature selection process; (**b**) The ordinate is the binomial deviation, and the abscissa is log(λ).

Among the 16 multiparametric ultrasound features, six predictors were associated with breast malignant tumors according to the Lasso regression. The candidate imaging biomarkers were “tumor shape”, “tumorDWR”, “tumor margin”, “tumor E-max”, “tumor E-ratio”, and “tumor MVDP” (Figure 5a,b). The formula to calculate the score was as follows:

$$\begin{array}{c} -22.95803+\left[1.67276*DWR\right]+\left[19.06989*Shape\right]+\left[1.05506*Margin\right]+\left[1.01657*E\text{-}max\right]+\\ \left[1.95513*E\text{-}ratio\right]+\left[2.35704*MVDP\right]. \end{array}$$

**Figure 6:**
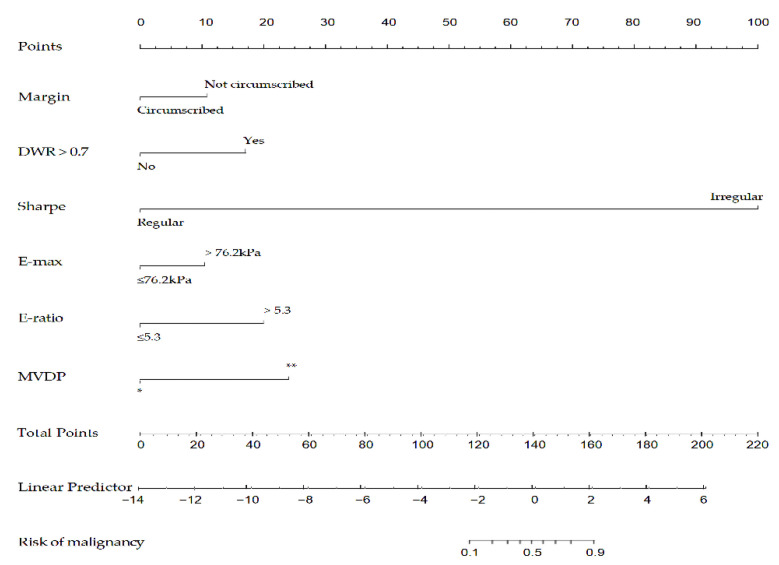
A nomogram to predict the risk of breast cancer for patients with breast lesions. DWR: depth-width ratio; E-max: Elasticity maximum; E-ratio: Ratio of Elasticity of lesion to normal tissue; MVDP: microvascular distribution pattern; *, non-vascular pattern, linear or curvilinear pattern and tree-like pattern; **, root hair-like pattern and crab claw-like pattern.

Afterward, the nomogram was developed based on the above six risk factors of breast cancer (Figure 6). According to this study, the nomogram’s sensitivity, specificity, positive predictive value (PPV), and negative predictive value (NPV) are 95.6%, 90.3%, 86.0%, 97.0%, respectively.

### 3.4. Validation of the Nomogram

**Figure 7:**
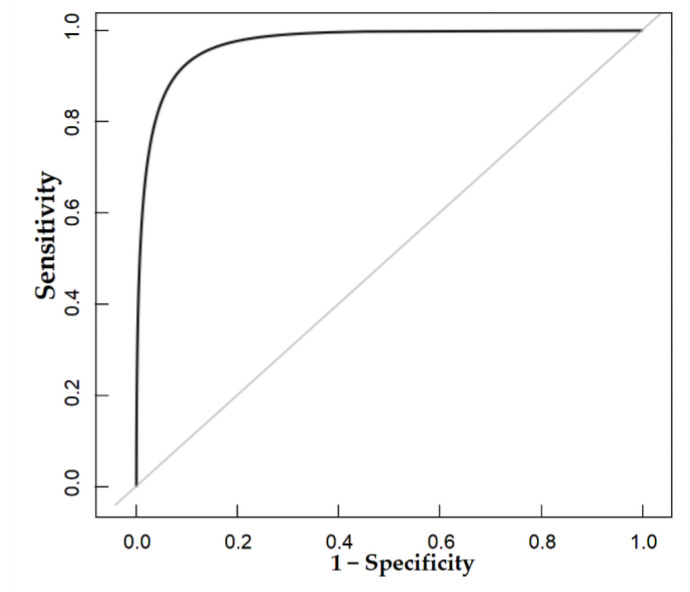
The AUC of the internal validation of our model.

**Figure 8:**
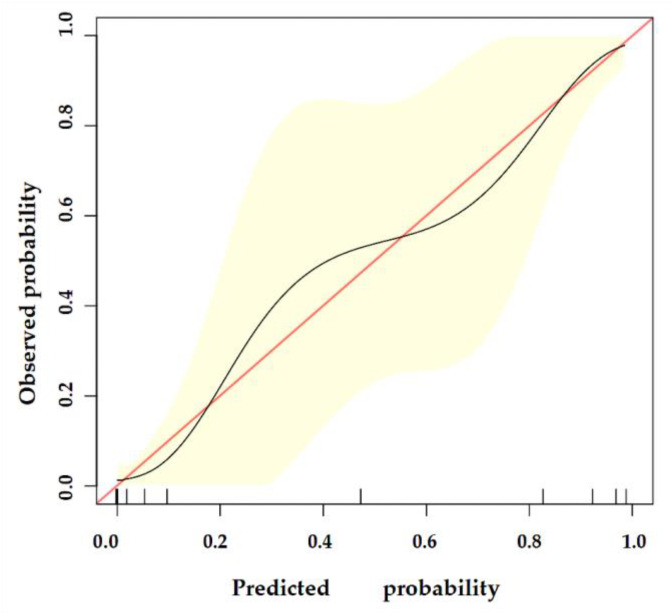
The calibration curve of our model. The y-axis represents the actual probability of breast malignancy, and the x-axis represents the observed probability. The red line represents the perfect prediction of an ideal model. The black line represents the predictive performance of our model, and the yellow shaded area represents our model’s 95% CI. Our model demonstrates good consistency between predicted and actual probabilities.

**Figure 9:**
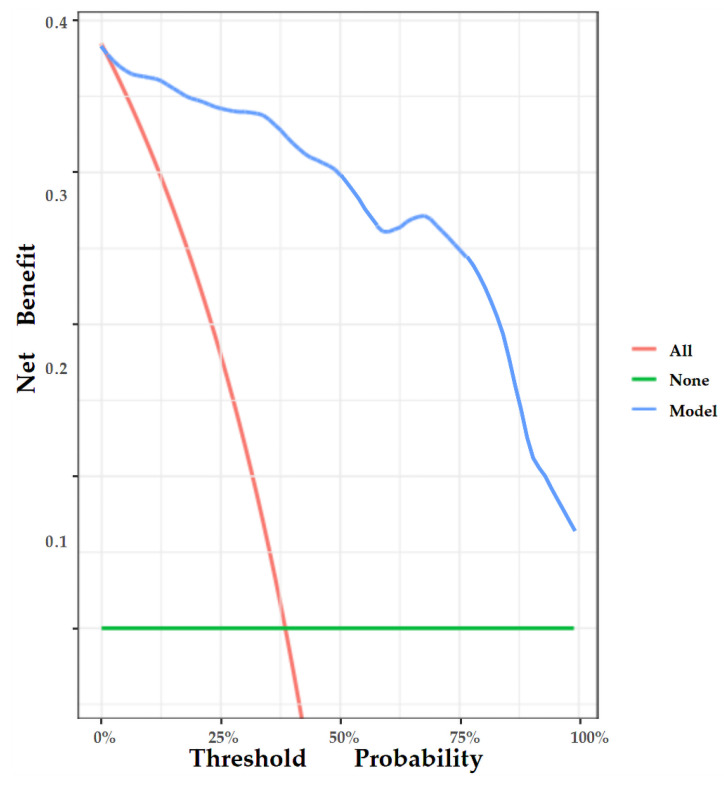
DCA of the nomogram. The y-axis represents the net benefit, and the x-axis represents the threshold probability. The red line represents the assumption that all patients tested positive and were all tested. The green line represents the assumption that all patients tested negative and all were not tested. The blue line represents the net benefit of our model as the threshold probability changes. It shows that our model can benefit patients with breast lesions across a considerable threshold range.

The nomogram was internally validated by 500 bootstrap samples to decrease the overfit bias. In addition, the nomogram for malignancy prediction exhibited an ideal AUC of 0.965, with −91.6% sensitivity, 91.1% specificity, 94.2% PPV, and 87.2% NPV (Figure 7). In addition, the calibration curve of our model showed a relatively satisfying result consistency between the predicted probability and the actual probability (Figure 8). As a final note, the result of DCA demonstrated that patients with breast lesions across a considerable threshold range can benefit from our model (Figure 9).

## 4. Discussion

In our current study, we first developed and validated a novel nomogram combining conventional ultrasound, AP, and SWE imaging biomarkers to predict malignancy in patients with breast lesions. Furthermore, the discrimination performance of our model was found to be relatively encouraging, and there was good agreement between the predicted and actual probability of breast malignancy according to the calibration curve. In addition, the nomogram was found to be clinically useful as per DCA.

Two-dimensional gray-scale ultrasound can obtain real-time morphological information of breast masses, which helps to distinguish between benign and malignant breast masses [28]. However, it suffers from relatively low specificity for detecting malignant breast lesions [29]. In addition, several studies have shown that the vascularity and stiffness of breast tumors are also important features in distinguishing malignant from benign breast tumors [30,31,32]. Consequently, the rationale for this study was that a nomogram focused on these aspects would help improve the prediction of malignant breast lesions, and this may be the reason for the relatively encouraging results.

Compared to a previous similar study using a combination of conventional ultrasound, AP, and SWE to differentiate breast tumors, our study achieved better diagnostic performance, and this may be attributed to the fact that we used both qualitative and quantitative parameters of SWE, whereas the other study only used qualitative parameters of SWE. In addition, they used seven color patterns of SWE to distinguish between benign and malignant breast masses, and these patterns are not easy to distinguish in clinical practice [33]. A previous study applied qualitative parameters of SWE such as E-max and E-ratio to distinguish benign from malignant breast masses, i.e., the same parameters as those used by us. However, they had a slightly lower diagnostic efficiency (AUC = 0.849) than our model (AUC = 0.973) [34]. It may be because they selected vascular index (VI) to assess breast tumor vasculature, while we chose to use MVDP, which has been shown to be a better imaging biomarker of diagnostic efficacy in previous work [35]. Additionally, they used ROI to measure the SWE parameters of breast lesions. However, in case of lesions with irregular shapes, covering the whole lesion with a round or rectangular ROI becomes difficult. On the contrary, we traced the entire breast mass and the machine automatically calculated the SWE parameters. We propose that this measurement method can theoretically reflect the SWE parameters of the breast lesions more truthfully.

Contrast Enhanced Ultrasound (CEUS) is a pure blood pool imaging technology that enhances the ultrasonic backscattering effect of intravascular contrast agents by the injected contrast agents; CEUS is widely used to assess the microvasculature of breast lesions [36]. The diagnostic performance of our model was similar to that of a previous study combining SWE and CEUS parameters in the diagnosing of breast tumors [37]. However, CEUS is a relatively invasive examination, and its extensive clinical application is limited by its relatively high cost and potential allergic reactions caused by the contrast agents. On the contrary, AP is a contrast agent-free, microvascular, ultrasound imaging modality. Hence, compared with this study, cost-effectiveness and non-invasiveness are two important advantages of our model.

BI-RADS is a classification system widely used to assess the likelihood of malignancy of breast tumors. According to the degree of suspiciousness of malignancy, lesions are divided into benign (category 2 and 3), indeterminate (category 4), and malignant (category 5) [28]. A needle biopsy is recommended for masses that are classified as category 4 or higher to clarify their nature. However, previous research has shown that more than half of these lesions are proven to be benign on pathological assessment of the biopsy samples [38]. Therefore, our research is significant because it has the potential to help sonographers choose the most valuable imaging biomarkers for diagnosis and to improve the differentiation of benign and malignant breast tumors for each patient based on disease likelihood and individually tailored assessments via the nomogram. We believe that using our nomogram will not only assist clinicians and patients in deciding whether needle biopsy should be performed or not, but also avoid unnecessary surgery in patients with benign breast lesions.

Some limitations of our study should be acknowledged. First, our study is a single-center, prospective cohort with a relatively small sample size, which limits the generalizability of the findings. Future large-scale, multi-center studies are needed to add to the database. Second, there are no external validation datasets available, although we performed the 500 bootstrap samples for internal validation. Hence, this prediction tool seems to have reasonable clinical utility; however, further validation in external datasets is necessary. Lastly, limited by the size of the sampling frame of the SWE image, we only included breast masses with the largest diameter of ≤ 30 mm, which may have introduced a selection bias. Further comprehensive studies are therefore required to resolve these issues.

## 5. Conclusions

In summary, we developed a nomogram based on SWE, AP, and conventional ultrasound imaging biomarkers to predict malignancy in patients with breast lesions. Furthermore, our model demonstrated relatively satisfactory discrimination between malignant and benign lesions as well as good calibration and clinical usefulness. Therefore, our nomogram can be a potentially useful tool for an individually tailored diagnosis of breast tumors in clinical practice.

## Figures and Tables

**Table 1:** Baseline characteristics of enrolled patients.

		Benign Group	Malignant Group	*p* Value
		72	45	
Sex				
	Male	0	0	NA
	Female	72	45	NA
Age (y)		39.0 (32.0–46.0)	43.0 (37.0–54.0)	0.008
Tumor Size (mm)		11.4 (8.9–15.9)	17.6 (12.1–23.2)	0.001
Shape				<0.001
	Regular	31	0	
	Irregular	41	45	
DWR > 0.7				0.005
	Absent	55	23	
	Present	17	22	
Margin				<0.001
	Circumscribed	36	5	
	Not circumscribed	36	40	
Echo pattern				0.021
	Hypoechoic	46	29	
	Isoechoic or hyperechoic	18	4	
	Heterogeneous	8	12	
Microcalcification				<0.001
	Present	18	26	
	Absent	54	19	
halo				
	Present	4	17	<0.001
	Absent	68	28	
Posterior features				0.007
	Enhancement	22	16	
	Shadowing	7	11	
	None	42	14	
	Combined pattern	1	4	
AG				
	Grade 0	10	1	<0.001
	Grade 1	32	5	
	Grade 2	14	14	
	Grade 3	16	25	
MVDP				
	Non-vascular	10	1	<0.001
	Linear or curvilinear	31	3	
	Tree-like	25	0	
	Root hair-like	5	12	
	Crab claw-like	6	29	
Stiff rim sign				
	Present	6	34	<0.001
	Absent	66	11	
E-max (kPa)		38.2 (28.6–46.5)	139.3 (92.3–205.1)	<0.001
E-max > cut-off (76.2)				
	Yes	7	36	<0.001
	No	65	9	
E-mean (kPa)		18.3 (13.7–22.8)	40.8 (26.4–60.5)	<0.001
E-mean > cut-off (25.4)				
	Yes	10	35	<0.001
	No	62	10	
E-min (kPa)		3.6 (0.5–8.5)	0.1 (0.1–0.9)	0.944
E-min > cut-off (12.9)				
	Yes	3	4	0.295
	No	69	41	
E-sd (kPa)		6.9 (5.2–8.3)	27.2 (15.7–42.2)	<0.001
E-sd > cut-off (14.6)				
	Yes	7	34	<0.001
	No	65	11	
E-ratio		1.8 (1.4–3.3)	7.2 (4.8–10.2)	<0.001
E-ratio > cut-off (5.3)				
	Yes	5	33	<0.001
	No	67	12	

Continuous variables that do not conform to the normal distribution were described by medians (inter-quartile range). AG: Adler Grade; DWR: depth-width ratio; MVDP: microvascular distribution pattern; E-max: Elasticity maximum; E-mean: Elasticity mean; E-min: Elasticity minimum; E-sd: Elasticity standard deviation; E-ratio: the stiffness ratio of the lesion compared to the surrounding adipose tissue calculated by placing two ROIs of 2 mm diameter at the same depth as that of the lesion.

**Table 2:** Pathological Findings of lesions.

Pathology Result	Number of Lesions (%)
Benign	72 (61.5%)
Adenosis	34 (29.1%)
Fibroadenoma	24 (20.5%)
Intraductal papilloma	7 (6.0%)
Chronic mammary inflammation	3 (2.6%)
Ductal epithelial hyperplasia	3 (2.6%)
Adeno-myoepithelioma	1 (0.8%)
Malignant	45 (38.5%)
Ductal carcinoma in situ	9 (7.7%)
Invasive ductal carcinoma grade 1	6 (5.1%)
Invasive ductal carcinoma grade 2	12 (10.3%)
Invasive ductal carcinoma grade 3	8 (6.8%)
Invasive lobular carcinoma grade 1	3 (2.6%)
Invasive lobular carcinoma grade 2	3 (2.6%)
Invasive lobular carcinoma grade 3	2 (1.7%)
Neuroendocrine carcinoma	2 (1.7%)

Data are presented as numbers (percentages).

## Data Availability

Not applicable.

## Abbreviations

AG: Adler grade; AP, Angio Planewave Ultrasensitive Imaging; AUC: area under the curve; BI-RADS: Breast Imaging Reporting and Data System; CEUS: contrast-enhanced ultrasonography; CI: confidence interval; CDFI: color Doppler flow imaging; DWR: depth-width ratio; E-max: Elasticity maximum; E-mean: Elasticity mean; E-min: Elasticity minimum; E-sd: Elasticity standard deviation; E-ratio: Ratio of Elasticity of lesion to normal tissue; MVDP: microvascular distribution pattern; PDI: power Doppler imaging; PV: penetrating vessel; ROC: receiver operating characteristic curve; SMI: superb microvascular imaging; VI: vascular index.

## Appendix A. Detailed Descriptions of Shear Wave Elastography Parameters

In SWE, ultrasound radiation forces are applied, inducing shear waves in tissue. The propagation of the shear wave (shear wave velocity “*v*”) correlates with tissue elasticity represented by Young’s modulus (E). Young’s modulus can be estimated by the following formula: E=3ρv2. Therefore, E-max is defined as the maximum value of the Young’s modulus of the mass. By analogy, E-mean is defined as the mean value of Young’s modulus of the mass and E-min is defined as the minimum value of Young’s modulus of the mass. In addition, E-sd was described as the difference between each value of Young’s modulus of the lesion and the E-mean. E-ratio was defined as the ratio of the value of the Young’s modulus within the tumor to the value of the Young’s modulus of the normal fat tissue at the same depth.

## Appendix B. Details of the R Software Package Used in Our Study

The “glmnet” packages were used for Lasso logistic regression. The “glm” packages were used for univariate and multivariate logistic regression. In addition, to construct nomogram and plot calibration curve, the “rms” package was used. ROC curves were plotted with the “pROC” package, and the decision curves analysis with the “rmda” package.
